# Supplementary figures and images for: BrassicaEDB: A Gene Expression Database for Brassica Crops
Source: Int J Mol Sci. 2020 Aug 13;21(16):5831. doi: 10.3390/ijms21165831 (PMC7461608; doi:10.3390/ijms21165831)

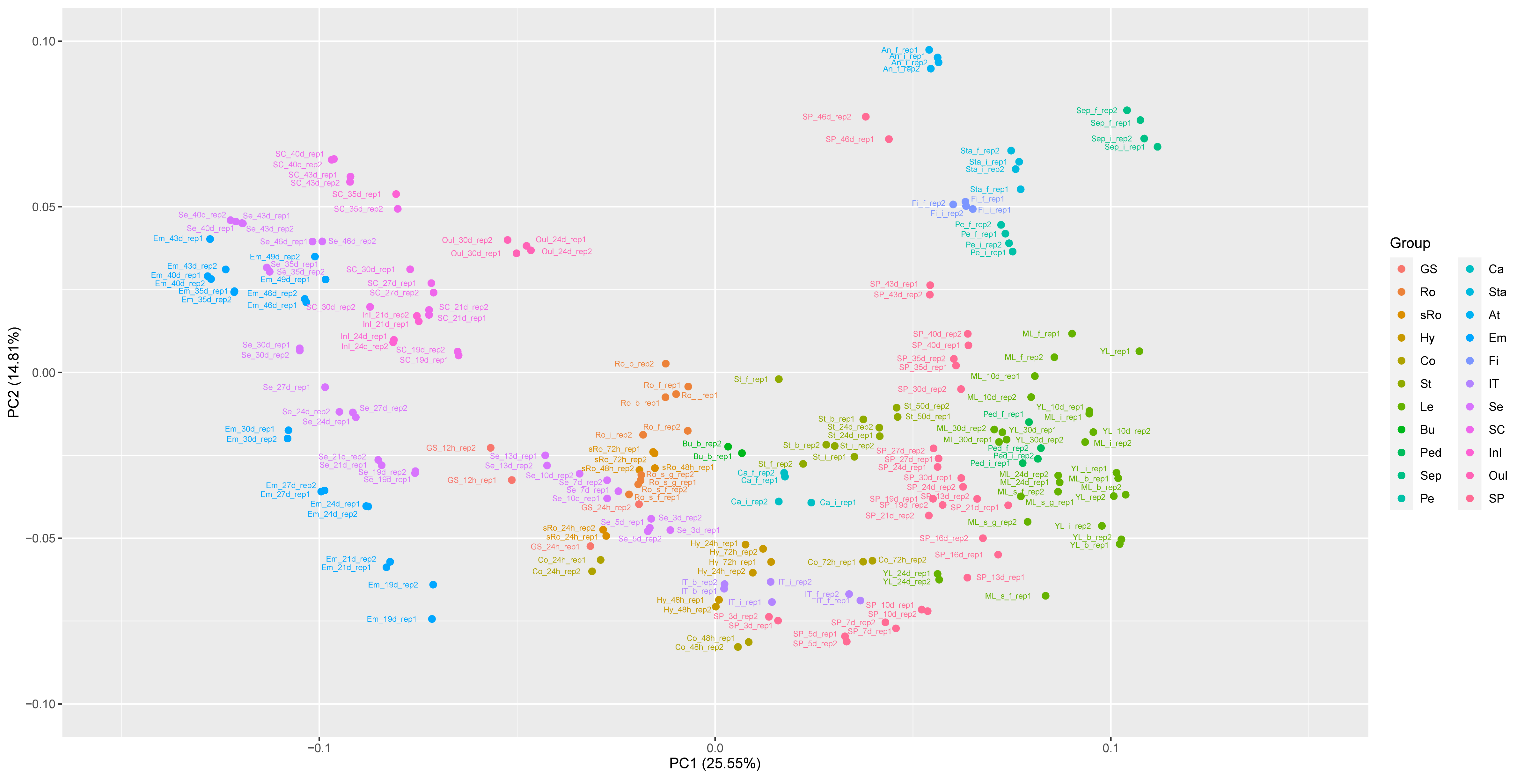

Supplement: Supplementary file 1 [file ijms-21-05831-s001.zip › Fig. S1.tif]

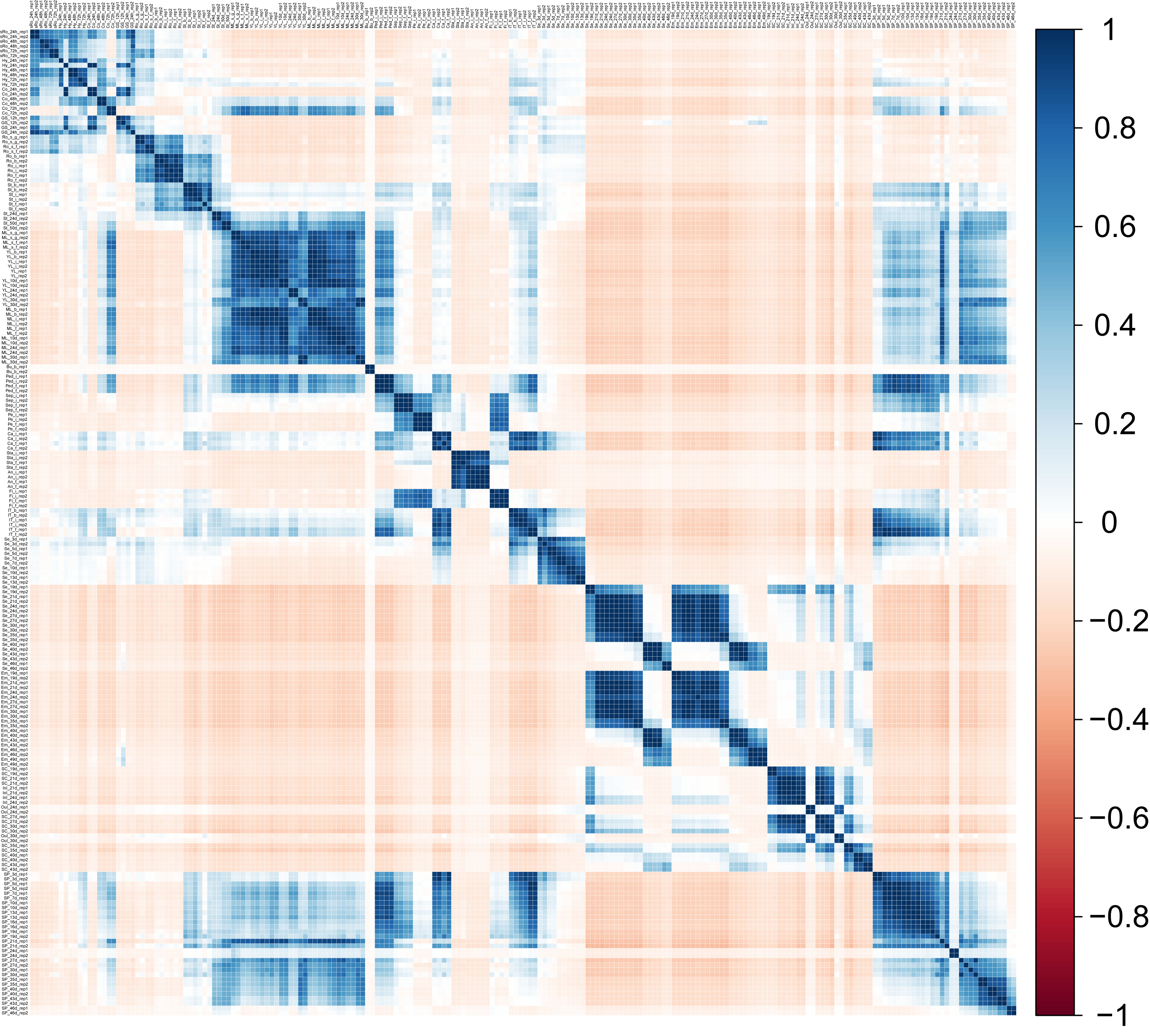

Supplement: Supplementary file 1 [file ijms-21-05831-s001.zip › Fig. S2.tif]

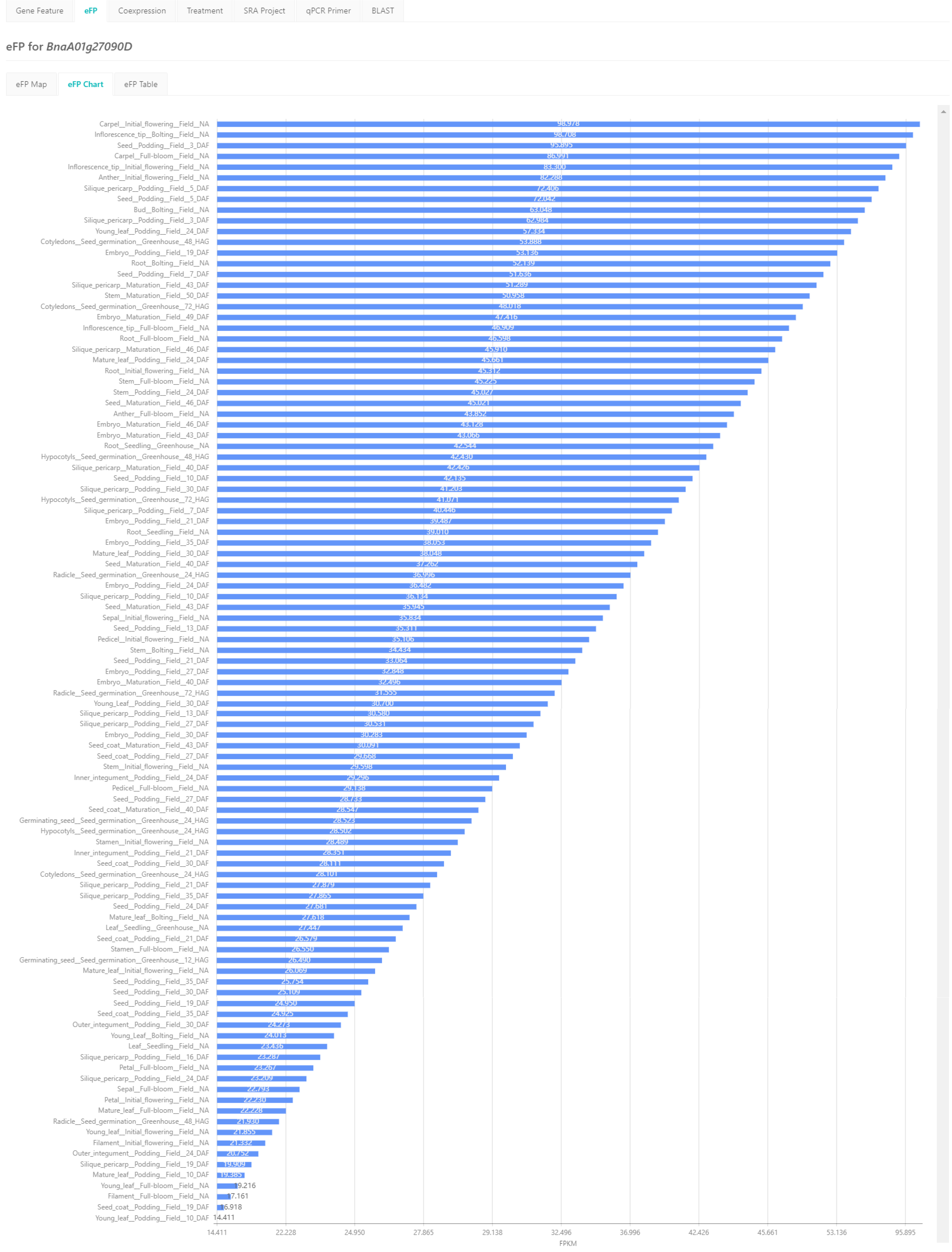

Supplement: Supplementary file 1 [file ijms-21-05831-s001.zip › Fig. S3.tif]

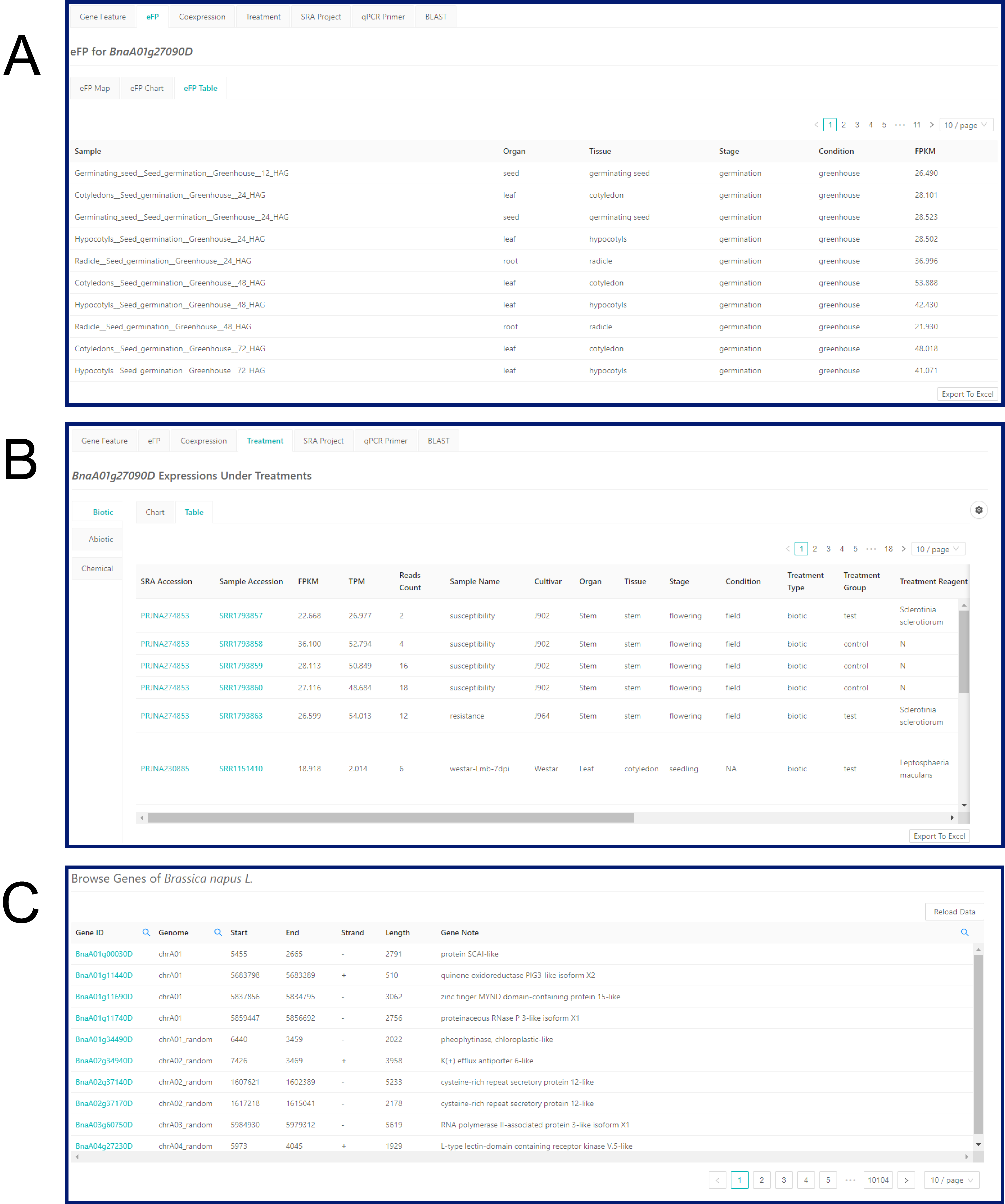

Supplement: Supplementary file 1 [file ijms-21-05831-s001.zip › Fig. S4.tif]
